# Investigating the contribution of image time series observations to cauliflower harvest-readiness prediction

**DOI:** 10.3389/frai.2024.1416323

**Published:** 2024-09-18

**Authors:** Jana Kierdorf, Timo Tjarden Stomberg, Lukas Drees, Uwe Rascher, Ribana Roscher

**Affiliations:** ^1^Remote Sensing Group, Institute of Geodesy and Geoinformation, University of Bonn, Bonn, Germany; ^2^Institute of Bio- and Geosciences, IBG-2: Plant Sciences, Forschungszentrum Jülich GmbH, Jülich, Germany

**Keywords:** explainability, deep learning, feature contribution, GroupSHAP, harvest-readiness

## Abstract

Cauliflower cultivation is subject to high-quality control criteria during sales, which underlines the importance of accurate harvest timing. Using time series data for plant phenotyping can provide insights into the dynamic development of cauliflower and allow more accurate predictions of when the crop is ready for harvest than single-time observations. However, data acquisition on a daily or weekly basis is resource-intensive, making selection of acquisition days highly important. We investigate which data acquisition days and development stages positively affect the model accuracy to get insights into prediction-relevant observation days and aid future data acquisition planning. We analyze harvest-readiness using the cauliflower image time series of the GrowliFlower dataset. We use an adjusted ResNet18 classification model, including positional encoding of the data acquisition dates to add implicit information about development. The explainable machine learning approach GroupSHAP analyzes time points' contributions. Time points with the lowest mean absolute contribution are excluded from the time series to determine their effect on model accuracy. Using image time series rather than single time points, we achieve an increase in accuracy of 4%. GroupSHAP allows the selection of time points that positively affect the model accuracy. By using seven selected time points instead of all 11 ones, the accuracy improves by an additional 4%, resulting in an overall accuracy of 89.3%. The selection of time points may therefore lead to a reduction in data collection in the future.

## 1 Introduction

Cauliflower cultivation is subject to high-quality standards. The optical appearance of cauliflower heads to be sold is crucial, which makes it essential to determine the exact time of harvest. However, the plants grow self-covered by their leaves, which makes determining harvest-readiness particularly difficult. Thus, an approach that contributes to determining the harvest time without harming the plants—e.g., by cutting away the leaves to see the heads—has a high value. Monitoring can be done through remote sensing techniques, such as unmanned aerial vehicles (UAVs; Chi et al., 2016; Weiss et al., 2020). The use of UAVs is significantly less resource-intensive than evaluating the plants from the ground by humans, as one image covers a large area of the field. At the same time, modern systems offer high resolution and image quality.

Determining harvest information of cauliflower from UAV images of single points in time has been done by Kierdorf and Roscher (2023). Opposed to individual time points, which can only capture a plant's current state to a limited extent, time series, enabling continuous monitoring of the entire growth cycle of plants, offer insights into the dynamic and current rate of plant development. This facilitates the comprehensive analysis of growth patterns and the estimation of crop yields. Utilizing image time series has already shown great success in the field of satellite data, e.g., for crop type mapping (Turkoglu et al., 2021; Rußwurm et al., 2023; Rußwurm and Körner, 2017) or yield prediction (Van Klompenburg et al., 2020; Schauberger et al., 2020; Yli-Heikkilä et al., 2022). Therefore, using time series shows a high potential for improving the accuracy of harvest prediction using UAV images.

UAV data acquisition and processing on a weekly or even daily basis is time-consuming. Sambasivan et al. (2021) have shown that the optimization through the reduction of low-quality data enables model improvement, as this data harms the result. Dodge and Karam (2016) have shown that low-quality data results, e.g., from time points that are less relevant or have no information gain for model predictions. Thus, finding time points that contribute most to a correct harvest-readiness estimation is crucial to improving the model and resources like time and money for future observations.

Deep learning (DL) methods leverage neural networks (NNs) to acquire complex patterns in data and enable automated analysis in the domain of plant phenotyping. Explainable machine learning (ML) techniques have been employed to select salient features that contribute to the decisions made by the NN (Chen et al., 2018; Mostafa et al., 2023; Harfouche et al., 2023) and can be divided in for example gradient-based methods (Simonyan et al., 2013; Springenberg et al., 2014; Smilkov et al., 2017; Selvaraju et al., 2017; Sundararajan et al., 2017) and perturbation-based methods (Zeiler and Fergus, 2014; Ribeiro et al., 2016; Lundberg and Lee, 2017; Petsiuk et al., 2018). Currently, explainable ML has primarily been applied to single images to derive pixel-wise information regarding feature attribution or significance in relation to the model's predictions (Uijlings et al., 2012; Gevaert et al., 2022). In plant phenotyping, explainable ML has been used to support tasks such as disease detection (Ghosal et al., 2018; Toda and Okura, 2019; Akagi et al., 2020; Wei et al., 2022) or plant classification (Grinblat et al., 2016; Desai et al., 2019). The application of explainable ML to image time series has predominately been performed for satellite data so far due to challenges in time-series analysis such as missing time series data, handling equidistant intervals between time points with UAVs (Drees et al., 2022; Kolhar and Jagtap, 2021), or unequal time-series length. Thus, most studies using explainable ML have focused on one-dimensional time series data only (Schlegel et al., 2019; Theissler et al., 2022; Villani et al., 2022; Shickel and Rashidi, 2020; Rojat et al., 2021), such as determining the importance of features in temperature or torque sequences (Siddiqui et al., 2019).

In our work, we classify cauliflower plants concerning their harvest-readiness using image time series showing plants and their development over time. For this, we use a modified ResNet18 (He et al., 2016) as a classifier. We compare models using images of single points in time shortly before harvest (Kierdorf and Roscher, 2023) to models using image time series with initial acquired time points without explicit selection. Furthermore, we use the explainable ML method GroupSHAP (Jullum et al., 2021) to investigate which image time points contribute most to the model's prediction. With this information, we selectively determine time points that increase the model's accuracy. We compare the time points with the respective development stages of the plants. From this, we conclude which developmental stages are generally important to determine harvest-readiness and propose how to reduce data acquisition resources.

The main contributions of this paper are as follows:

- Utilizing time series data, as opposed to single time points, enhances the predictive accuracy of cauliflower harvest-readiness by up to 4% through the integration of developmental information.
- Applying GroupSHAP for selecting specific time points, especially in leaf and shoot development interval, further increases accuracy by an additional 4%, reaching up to 89%. This method aligns with growth stages and offers the potential for reducing resource requirements in future data acquisition efforts.

## 2 Materials and methods

### 2.1 Data

We use image time series data from field 2 of the GrowliFlowerR dataset (Kierdorf et al., 2022), showing the development of cauliflower from planting to harvest. The dataset contains information about planting and harvest day for each cauliflower plant. The planting day is used to derive the day after planting (DAP) for each image in the time series, which represents the age of the plant. Harvesting took place on four dates. The images in the dataset are georeferenced and have the same resolution and scale. Due to different weather conditions at different DAPs, factors such as exposure and soil irrigation differ at various points in time.

**Figure 1:**
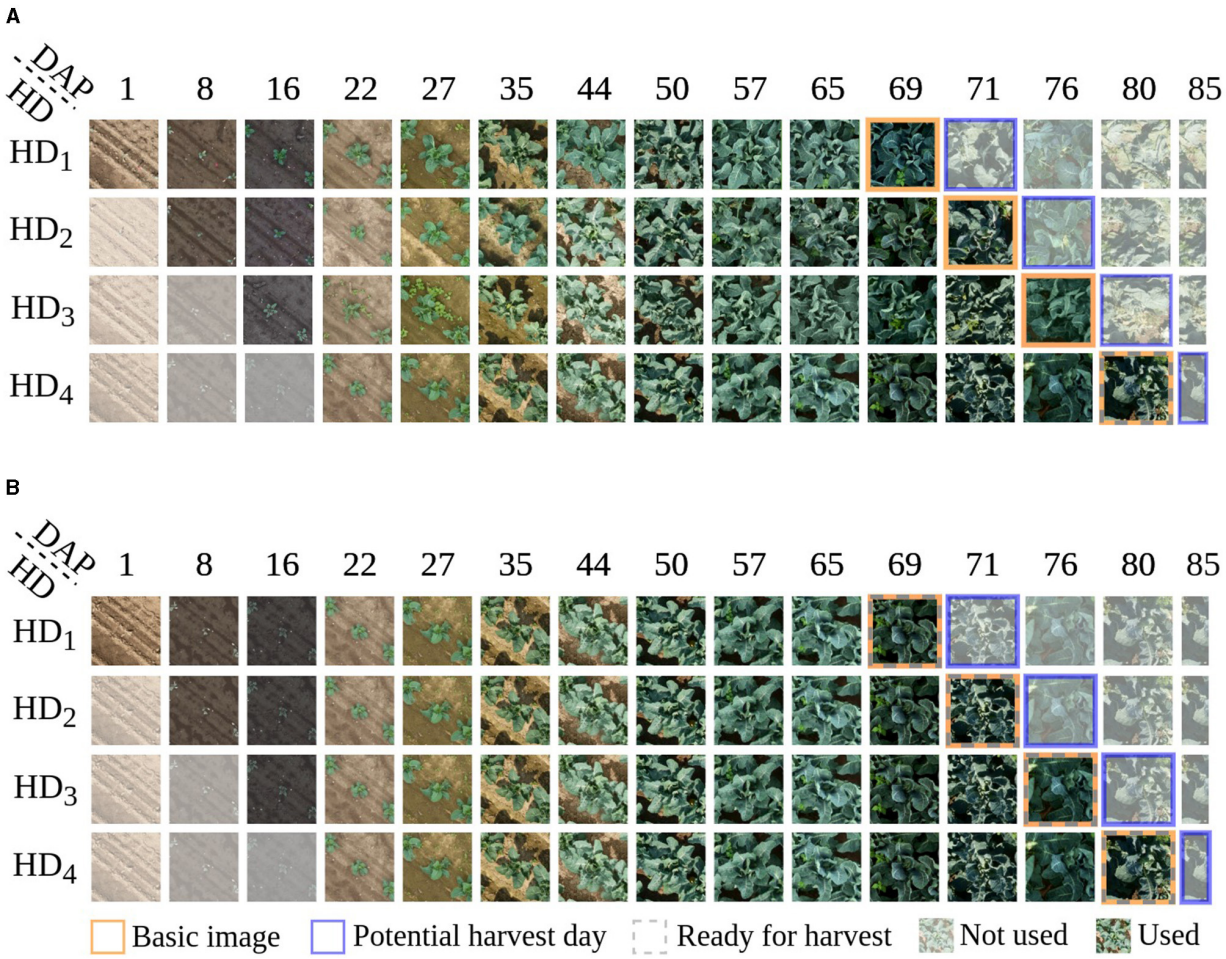
Visualization of cauliflower image time series, with a length of *T* = 11, presenting various potential harvest days HD, indicated by the blue frames, whereby a row illustrates an individual time series. **(A)** shows an example of how to generate time series for an individual plant. In this example, a plant is observed and labeled as Not-ready for harvest on days HD_1_, HD_2_, and HD_3_, shifting to Ready for harvest on harvest day HD_4_, indicated by the gray dashed frame. Each time series is shifted by one image for HD_1_ to HD_4_, reflecting the progression over time. The variability in potential harvest days results in differences among the basic images within the time series, indicated by the orange frame. Consequently, the corresponding images at days after planting (DAP) also shift accordingly. Only non-transparent images are utilized as input for constructing the time series. For plants deemed Ready for harvest on HD_1_, there exists only one plausible time series since harvesting occurs on that specific day. Equivalently, for plants harvested on HD_2_ and HD_3_, there are two and three conceivable time series, respectively. This method of generating time series remains applicable across varying lengths of time series. **(B)** shows time series of four different plants representing the four harvest days. This illustration is used to compare Ready and Not-ready for harvest plants.

The data used for this work is selected and processed in the same way as the data for the work of Kierdorf and Roscher (2023), who deal with harvest-readiness prediction based on single time points. They use images right before harvest, as shown in Figure 1 highlighted in orange, and divide them into classes Ready and Not-ready for harvest. We refer to these images as basic images. For our time series classification approach, we extend the basic images by *T*−1 images acquired chronologically before the used basic image, resulting in a time series with *T* individual time points. Each image within the time series represents a different developmental stage of the plant. We vary *T* for later experiments with *T*∈{1, 2, …, 11}, resulting in time series with different temporal lengths. We denote these time series as initial time series (iTS). We use the training, validation, and test set as described in Kierdorf et al. (2022) and apply standard augmentations according to Kierdorf and Roscher (2023) like flipping and rotation on the training data. This involves augmenting the entire image time series of a plant with the same augmentations. After data augmentation, the training set contains 6,224 time series, 2,432 of class Not-ready and 3,792 time series of class Ready. The validation set and the test set consist of 196 and 194 time series each.

Example image time series with a length of *T* = 11 for one specific plant are shown in Figure 1A. The presented plant is observed and labeled as Not-ready for harvest on harvest days HD_1_, HD_2_, and HD_3_, shifting to Ready for harvest on harvest day HD_4_. Each time series corresponds to one of the harvest days. If a plant is classified as not ready for harvest on a given harvest day, it is reclassified for the next harvest day. The variability in potential harvest days results in differences among the baseline images within the time series, indicated by the orange frame. As we align the time series with these baseline images, the temporal start and end points of the series shift toward the harvest day, depicted by non-transparent images. For plants deemed Ready for harvest on HD_1_, there exists only one plausible time series since harvesting occurs on that specific day. Equivalently, for plants harvested on HD_2_ and HD_3_, there are two and three conceivable time series, respectively. This method of generating time series remains applicable across varying lengths of time series. Thus, we can generate up to four time series for a specific plant, dependent on the harvest day. The images are aligned by DAP and illustrate which DAP is used for classification regarding the potential harvest days. Since the basic images were taken on different DAPs depending on the potential harvest day, iTS contain different stages of development. Figure 1B compares four different plants, each labeled with a different harvest day.

Available developmental stages of cauliflower according to Feller et al. (1995) are listed in Table 1. We start with listing stage 12, as the plants were planted in the field out of seedling trays and consist of two or more leaves at the point of planting (Kierdorf et al., 2022). The developmental code comprises the macro stage (first number) and the micro stage (second number). Important stages for cauliflower are macro stage 1 “Leaf development (main shoot)” and macro stage 4 “Development of vegetative plant parts (harvested material).” We set the mean head size per HD concerning the DAP, illustrated in Table 2. The colors represent the different developmental stages listed in Table 1. We see that certain stages of development spread over several flight dates. On average, the harvest-ready plants on different HDs develop at different speeds. Particularly shortly before harvest, major variations can be seen between the HDs. Although the development is spread out, there is a certain correlation between development and acquisition day.

**Table 1:** BBCH developmental stages on the field for cauliflower according to Feller et al. (1995).

**Code**	**Explanation**
12	2. leaf unfolds
13	3. leaf unfolds
1x	Stages consecutive to...
19	9 or more leaves unfold
2x	Not available for cauliflower
3x	Developing the main shoot
40	Start of flowering
41	Start of flowering: Vegetation cone width > 1cm
43	30% of the expected head diameter is reached
45	50% of the expected head diameter is reached
47	70% of the expected head diameter is reached
48	80% of the expected head diameter is reached
49	Species/variety-typical size and shape achieved;
	head still firmly closed

The code represents the developmental stage and is made up of the macro stage (first number) and the micro stage (second number). The expected head diameter for cauliflower is about 15 cm. The colors are used to set the code in relation to the acquired data in Table 2.

**Table 2:** Overview over the mean head size per harvest day (HD) per day after planting (DAP).

	**Mean head size [cm]**			
**DAP**	HD_1_	HD_2_	HD_3_	HD_4_
44	0.9	0.7	0.4	0.1
50	0.9	0.8	0.5	0.2
57	2.1	1.9	1.6	1.2
65	7.7	6.1	4.6	3.3
69	10.9	8.8	6.7	5.6
71	-	12.0	9.4	7.3
76	-	-	13.5	10.0
80	-	-	-	12.2

The colors represent the different developmental stages shown in Table 1. The numbers are calculated based on the *in-situ* measurements of GrowliFlower dataset (Kierdorf et al., 2022).

### 2.2 Classification network

**Figure 2:**
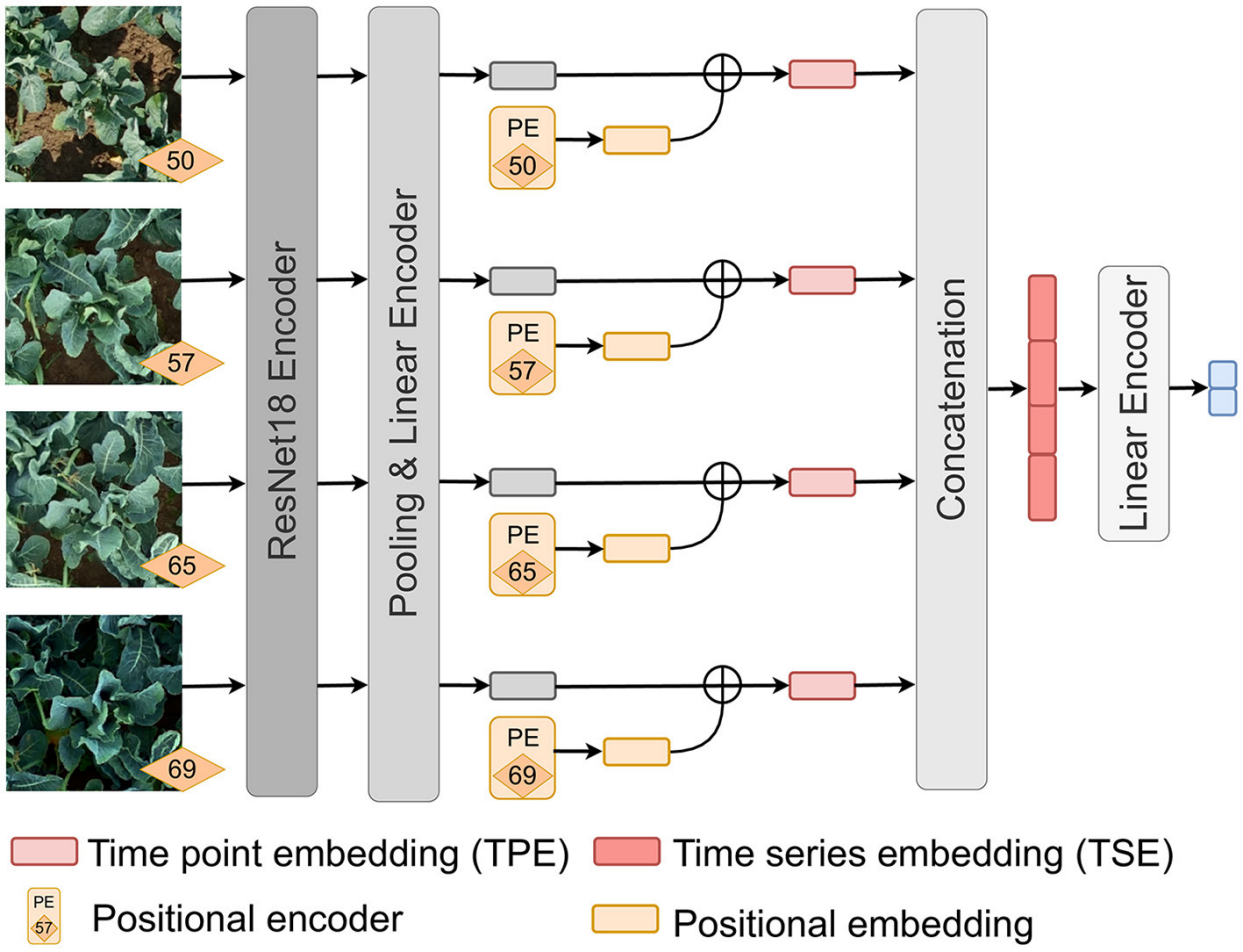
Network for cauliflower image time series classification. Each image of a time series is fed into the network successively. The weights of the network are updated after the entire time series has passed through the network.

Our model (see Figure 2) is used to classify RGB image time series into two classes Ready and Not-ready for harvest. Each image of the time series is sequentially fed into the same ResNet18 (He et al., 2016) encoder, where the weights are updated only after the entire time series has passed through the network. To obtain a lower-dimensional feature embedding vector that can be used for explainable ML methods, we modify the size of the last standard fully connected layer within the encoder to 32. We add a positional encoding (Gehring et al., 2017) of the plant's age to the embedding. This adjustment allows for better differentiation between young plants and poorly developed plants. We refer to the resulting embedding as the time point embedding (TPE). The TPEs are then concatenated to form a time series embedding (TSE), which is fed into a linear encoder consisting of two linear layers to calculate the final scores for each class. The input dimension of the first linear layer in the encoder is equal to the length of the TSE (*T*×32). The output dimension is optimized by hyperparameter tuning based on the length of the time series *T* to retain most of the information. Therefore, the output dimension is defined by dividing the TSE length by a scaling factor λ. We have observed that this additional layer significantly improves the classification accuracy for time series.

In our architecture, each time series must have the same length, unlike vision transformers (Dosovitskiy et al., 2020) that can handle varying time series lengths (Garnot et al., 2020). However, our architecture has the advantage of requiring less data and fewer parameters to train an accurate model.

### 2.3 Shapley additive explanations

Shapley additive explanations (Lundberg and Lee, 2017) (SHAP) is a model-agnostic explainable machine learning method. SHAP is used to calculate the contribution of an entity to a model prediction where an entity consists of one or more features. The original SHAP approach (Lundberg and Lee, 2017) uses single features, while the GroupSHAP approach (Jullum et al., 2021) considers multiple features within an entity. In our work, we compute GroupSHAP values by defining an entity consisting of a combination of all features within a TPE. Thus, this entity represents the embedding of an input image of a time series. In doing so, we investigate the effect of individual time points on the model's accuracy rather than model features.

In general, an entity with a positive SHAP value contributes positively to a prediction and, thus, increases the model score, while an entity with a negative value contributes negatively and, thus, reduces the model score. A SHAP value represents the deviation from the mean contribution of an entity to the final prediction. To determine the SHAP value, first, all possible entity combinations are formed, where one of these combinations is referred to as a coalition. The entities within a coalition are fixed. Entities not present in a coalition are filled with random examples of the same entity from the training set to maintain a uniform number of entities required for neural networks. Afterward, the SHAP value is determined by computing the mean of differences between all coalitions, excluding the entity of interest, compared to the same coalitions, including the entity of interest. We calculate the weighted average over all coalition differences using a similarity measurement of the data samples, e.g., by using a kernel function such as Gaussian kernel or binomial coefficients. The resulting value gives the SHAP value for the entity of interest. Coalitions that consist of either only fixed entities or non-fixed entities are given the highest weight, as they are most likely to be used to derive direct entity contributions of the entities of interest. This process is carried out for all entities representing the different TPEs of a time series. The final prediction of a data sample is obtained by adding the SHAP value to the mean prediction of the entire dataset. In general, SHAP values are calculated for each target.

One issue to consider when using GroupSHAP is the assumption of feature independence, in our case, the independence of the embedding vectors. In real-world scenarios, features are often correlated, leading to misleading interpretations.

### 2.4 Experimental design

We present two experiments. For each experiment, we train one model for each input time series length *T*. We normalize the input images before feeding them into the model. The training for each model consists of at least 60 epochs and stops if validation accuracy does not increase significantly over 10 epochs. We use a batch size of 16 and the Adam optimizer with a learning rate of 1e − 5. The learning rate is reduced using a scheduler with a step size of 20 and a factor γ of 0.1. We adjust each model's weight decay and linear layer mentioned earlier through hyperparameter tuning. We consider weight decays α in the range of [1e − 1, 1e − 3] and scaling factors λ in the range of [2, 4]. As the final model of training, we select the model with the highest validation accuracy. For reproducibility, we set all used seeds to 0. We run our experiments on an AMD EPYC 7742 64-Core processor and an NVIDIA A100 PCIe graphic card with 40 GB hBM2 RAM. The model's runtime with the most input features with *T* = 11 is 14 min.

#### 2.4.1 Time series classification based on initial time series

For classification based on single time points, we assume that the time interval between image acquisition and harvest must be kept short, as factors such as weather still change the development considerably (Tollenaar et al., 2017). No prior knowledge about previous plant development is given in this case. We want to investigate whether the use of time series information for the classification of harvest-readiness is more beneficial than the use of individual time points because the use of time series integrates the temporal development of the plants into the model. We also address whether it is worth integrating early acquisition times to increase model accuracy or whether it is sufficient to use time points close to harvest.

For this purpose, we compare three types of models. In the first model, we use our designed model structure and single time points as input and denote this model as our baseline. As a reference to our baseline, we use the original ResNet18 model without an additional linear layer, which is also used by Kierdorf and Roscher (2023) for cauliflower harvest-readiness classification also based on single time point inputs. For both models, we use the basic images as input. As the third model type, we use our designed network and iTS as input. For all model types, we calculate the overall (oaAcc) and balanced class accuracy (bcAcc)


(1) $$\begin{array}{l} \text{bcAcc}=\frac{\frac{\text{TP}}{\text{TP}+\text{FN}}+\frac{\text{TN}}{\text{TN}+\text{FP}}}{2}, \end{array}$$

also known as the macro-average of recalls, and compare them across the different types of models. Equation 1 is composed of the true positives (TP), true negatives (TN), false positives (FP), and false negatives (FN) from the confusion matrix.

#### 2.4.2 Classification of time series based on the selection of time points with GroupSHAP

In this experiment, we investigate how single time points within a time series contribute to the classification result and how excluding time points affects the model accuracy. Literature has shown that excluding features (here time points) based on feature selection can improve the model accuracy (Bratu et al., 2008; Chu et al., 2012; Zou et al., 2015). We connect the time points with the BBCH developmental stages according to Feller et al. (1995) and investigate whether certain developmental stages have a low contribution to model accuracy and can, therefore, be omitted from data collection to conserve resources.

We begin by taking the iTS model with *T* = 11 calculated in our first experiment and (i) calculate the entity contribution of the time points using GroupSHAP. Then, we (ii) exclude the time point with the lowest mean absolute GroupSHAP from iTS over all harvest days since it has the most neutral contribution (closest to 0). In theory, the day with the lowest contribution would have to be excluded separately for each HD to receive the highest model accuracy, as different DAPs are contained in the time series of the different HDs. In practice, however, concerning resource-saving data acquisition, not only selected parts of the field are flown over, but the entire field, so that certain points in time must be completely excluded. Therefore, we exclude the time points with the lowest mean absolute contribution across all time points and, thus, exclude the mean macro developmental state over the whole field. We denote the new time series with the selected time points as selective time series (sTS). Next, we calculate (iii) a new model using the sTS and recalculate the accuracies. We repeat (i)–(iii) using the most recently determined sTS instead of iTS.

We specify that the first three and last four acquisition days are always included in the time series. Keeping the last four acquisition days is important because it allows us to determine whether the class Ready or Not-ready for harvest can be derived in the coming days. Without these time points, there is no reference point for predicting harvest-readiness. If we classify a plant as Ready, it will be ready for harvest within the coming days, i.e., the last image in the time series is the last one before harvesting. Including the time points close to harvest has proven to be beneficial in maintaining stable results despite weather fluctuations. Another reason for always including these seven time points is to minimize data bias toward a specific HD and maintain similar data for all models, we only consider time points for exclusion where an image can be excluded from each HD. The days that are excluded show on average the same developmental stage per time point (see Table 2). Different plant developments average out over the entire field. We assume that this will also be the case for the following growing seasons. For the experiment, fixing the seven time points allows only the calculation of sTS for time series length *T*∈[4, 10].

## 3 Results

### 3.1 Time series classification based on initial time points

**Figure 3:**
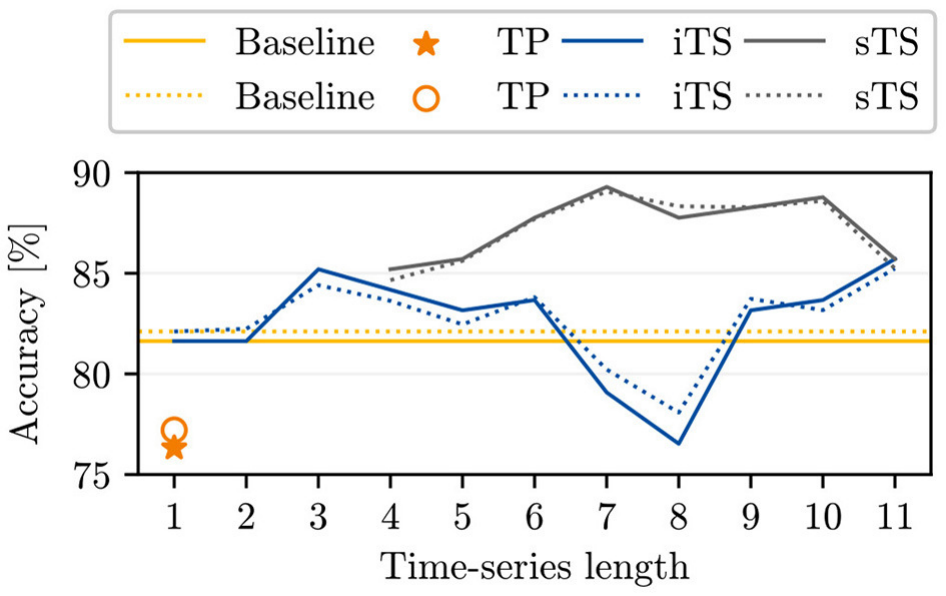
The plot opposes the baseline model accuracies for single time point inputs to the accuracies achieved by Kierdorf and Roscher (2023) (TP), to our computed initial time series (iTS) accuracies, and to the selective time series (sTS) accuracies. The plot shows the overall (oaAcc) and balanced class accuracy (bcAcc) for different time series lengths. For sTS, time points are excluded starting from right to left.

The comparison between the accuracies of our baseline and the reference for single time point classification as reported by Kierdorf and Roscher (2023) indicates that incorporating an additional linear layer into the model leads to an overall improvement in the achieved accuracies for single time point inputs (see yellow lines in Figure 3 compared to orange markers). Additionally, we find that using iTS input data generally enhances model accuracy compared to the baseline in nearly all cases. When adding successive time points, we observe higher accuracies for seven out of ten time series models, similar accuracy in one case, and lower accuracies in two cases compared to our baseline. The increasing trend in accuracy is initially noticeable, decreases between *T* = 4 and *T* = 8, and then rises again at *T* = 9 to reach a maximum value of 85.7%. The maximum increase in accuracy compared to the baseline is ~4% for a time series length of *T* = 11. By examining the time points added for specific time series lengths, we find that time points within the DAP interval [44, 65] are more likely to harm the accuracy.

### 3.2 Classification of time series based on the selection of time points with GroupSHAP

In comparing the sTS and iTS model accuracies, higher accuracies are consistently achieved across all time series lengths for sTS (see Figure 3). Compared to the best iTS model with *T* = 11, the sTS model accuracies maintain a similar or higher level with the exclusion of selective entities. The oaAcc and bcAcc for sTS models reach their maximum values of 89.3 and 89.1%, respectively, for a time series length of 7. However, the sTS model accuracies are lower for shorter time series (< 6TPs). Additionally, we observe that the sTS of length 5 achieves the same accuracy as the use of 11 initial time points.

**Figure 4:**
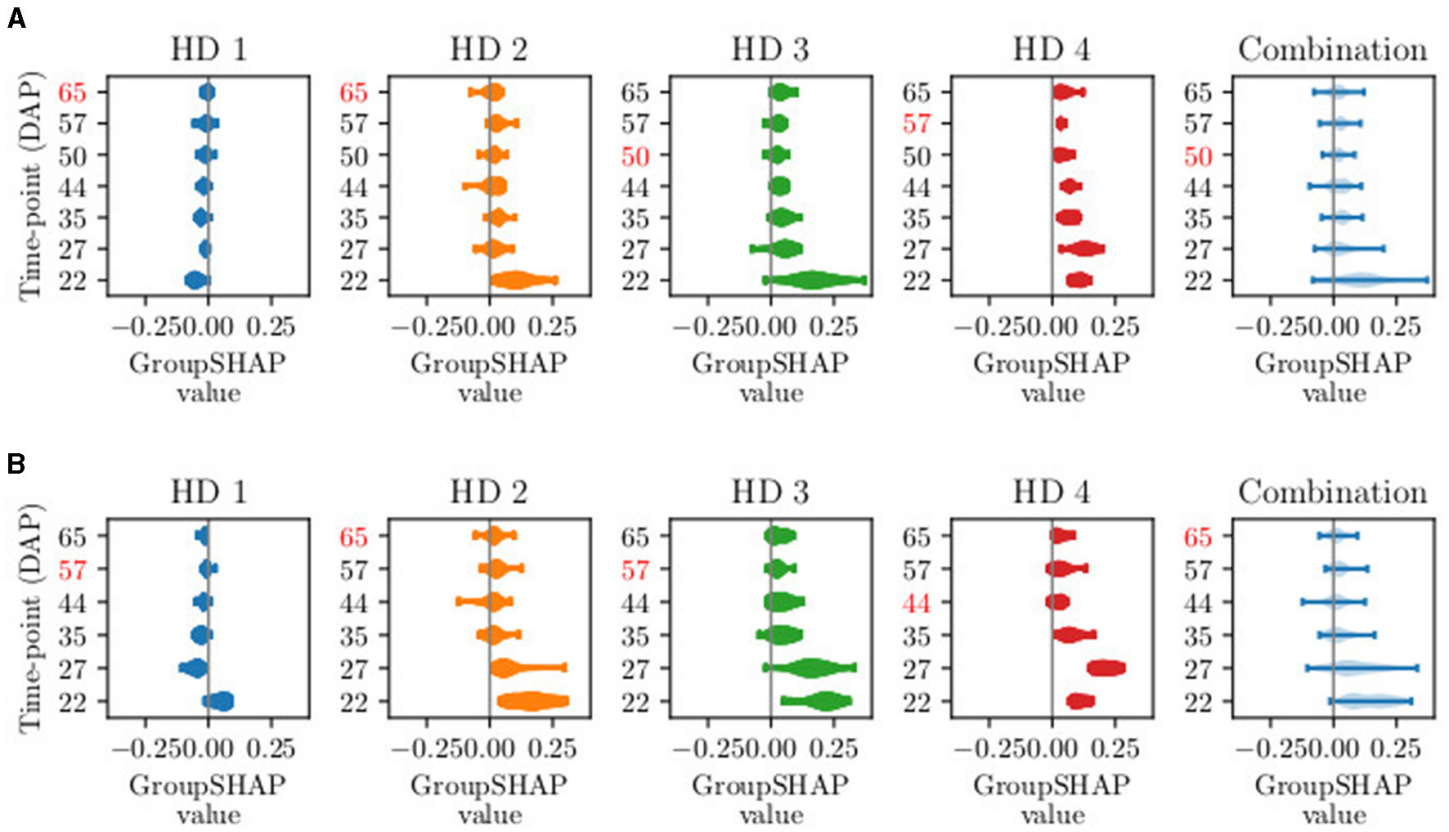
Visual example of GroupSHAP values for time series lengths *T* of **(A)** *T* = 11 and **(B)** *T* = 10. The fixed time points are not shown, as they are not excluded. One violin plot shows the distribution of GroupSHAP values per time point, more explicitly per day after planting (DAP). The first four plots represent the set of GroupSHAP values classifying data of harvest day (HD) 1–4. The light blue plot represents the combination of the four sets. The red-marked DAPs represent the days with the lowest mean absolute GroupSHAP value. The red-marked number in the combination plot is excluded in the next selective time series model.

Figure 4 presents the distribution of GroupSHAP values for sTS lengths with *T*∈[10, 11]. For a more detailed analysis, the GroupSHAP values are separated for potential harvest days, with a combined overview of all harvest days shown in light blue. All five plots are related to the class Ready for harvest. Regardless of the time series length, the model tends to classify more plants as Ready for harvest on later potential harvest days, reflecting the increasing number of plants ready for harvest over time. This trend is consistent even for shorter time series lengths. In practice, since data collection involves surveying the entire field, it is practical to eliminate data from an entire acquisition day. Therefore, we focus on the combination plots when considering the exclusion of time points. It turns out that on average, for *T* = 11, DAP 50 (macro stage 40) should be excluded, and for *T* = 10, DAP 65 (mean macro stage 43) should be excluded. The subsequent order for exclusion of DAPs is 57, 44, 22, 27, and 35.

To sum up our observations, we obtain the best model using sTS with 7 time points within a time series with oaAcc of 89.3% and bcAcc of 89.1%. We achieve an oaAcc of 76.3% and bcAcc of 76.7% with the same model on a test set. Weighing the effort of data acquisition against achievable model accuracy, we achieve an oaAcc of 85.2% and bcAcc of 84.7% on validation data and oaAcc of 78.9% and bcAcc of 78.9% on the test set when using 4 time points.

## 4 Discussion

### 4.1 Time series classification based on initial time points

We demonstrate that incorporating time series information enhances the predictive accuracy of the model, even when the cauliflower curd is not visible in any image within the series. Kierdorf and Roscher (2023) have demonstrated that it is possible to determine harvest-readiness even when the curd is occluded by the canopy. Using explainable machine learning through the Gradient-weighted Class Activation Mapping (Grad-CAM) interpretation technique, they have revealed that the ResNet18 model's decisions are influenced by specific image features, primarily the leaves at the center of the plant, which protect the curd. Given that our model also utilizes a ResNet18-based architecture, we expect these insights to be applicable to time series data as well. Furthermore, information on the plant's development over time provides additional features that enhance the prediction of harvest-readiness, thereby increasing accuracy.

We attribute the decrease in accuracy for iTS to the fact that not every time point in the dataset provides relevant information to the model. Some time points may exhibit redundancy or correlation, thereby sharing their contribution to the output. This could generally occur because there is no significant visual growth of the plants between two acquisition days. Particularly in the later stages of development, the plants may no longer grow visibly but continue to develop the head internally. Another reason could be that additional time points negatively impact the accuracy by confusing the model. This may result from irrelevant features or noise in the data (Dodge and Karam, 2016), such as slightly blurry images, which occur during the processing of raw data into orthophotos (Kierdorf et al., 2022). The subsequent increase in accuracy can be attributed to the inclusion of new informative features by adding additional images.

### 4.2 Time series classification based on initial time points

The improved accuracies for sTS demonstrate that GroupSHAP effectively selects relevant entities to enhance model accuracy. However, excluding too many features can result in losing valuable information essential for accurate predictions. In our case, this is because the approach uses the criterion of the lowest mean absolute value for exclusion. However, the lowest mean absolute value can also positively contribute to the predictions. This implies that, beyond a certain point, its exclusion leads to a decrease in accuracy. The exclusion of entities, therefore, only makes sense up to a certain point and has to be limited to maintain and achieve the best model accuracy. In addition, we have identified that shorter time series including selected time points yield similar accuracies compared to longer time series without time point selection. This suggests that feature selection can reduce time and costs in data acquisition and processing while achieving the same results as acquiring data over the entire growing period.

From a biological perspective, the images that are sorted out first show plants that are in the phenological development of macro stage 4 and micro stages 1–3, according to BBCH by Feller et al. (1995)). During these stages, head development takes place, and the head starts growing, reaching a diameter of up to 6cm. In the corresponding image data, there are minimal visual changes compared to earlier images, as the growth happens internally within the plant. The current appearance of the plant, which is used in the model's decision-making, is therefore determined from the images that display the most robust plant development. In contrast, the developmental stages of the days with the highest contribution (DAP 22, 27, and 35) are at the beginning of macro stage 3, when the main shoot begins to develop. Examining the first two excluded acquisition time points, DAP 50 and 65, we observe that they occur more frequently in the database for sTS models of length 7 and 8. The frequent presence of these acquisition days may explain the decline in the iTS curve as well. For iTS, we hypothesize that certain time points negatively impact accuracy due to irrelevant features or noise in the data, such as slightly blurry images. This insight also applies to sTS models and might further explain the low contribution of these time points.

It is important to note that our statement regarding the order of exclusion of DAPs may change depending on the development of the plants in response to external conditions. If the field develops, on average, one week earlier, this shift would apply to the entire field, resulting in a corresponding adjustment of all harvest days and development stages. When generalizing to other fields and farms, it is important to consider the development stages rather than solely relying on the DAP time point. Although we have not yet tested the trained model on another cauliflower farm, preliminary results indicated that the available data in the field for cauliflower harvest-readiness estimation is insufficient to generalize and transfer the classification model to other fields. The effects of varying weather, lighting, and irrigation must be accounted for to ensure generalizability. However, altering colors in the HSV color space to simulate changes in exposure and soil conditions can inadvertently modify the perceived biological properties of the plants. For instance, a color change might make healthy leaves appear diseased, or conversely, diseased leaves appear healthy.

GroupSHAP provides valuable insights but has several limitations that need to be addressed. One primary constraint is its high computational complexity and substantial processing time. The method evaluates the contribution of each feature across numerous permutations, which results in considerable computational demands, especially with large datasets and complex models. To mitigate this issue, parallel computation using multiple GPUs can be employed. Distributing the computational workload across several GPUs can significantly reduce processing time. Additionally, GroupSHAP is sensitive to data quality. The explanations generated by GroupSHAP rely heavily on the quality of the input data. Noisy, incomplete, or biased data can lead to incorrect attributions and interpretations. However, GroupSHAP can also help identify such issues, as features with poor quality will contribute minimally to the final prediction. Ensuring high data quality from the beginning thorough data cleaning and validation is essential to achieving accurate and reliable results.

## 5 Conclusion

In this work, we classify image time series of cauliflower plants, depicting the temporal development concerning their harvest-readiness. For this purpose, we use a ResNet18 model as an encoder and integrate the plant age through positional encoding to improve the discrimination between young and underdeveloped plants. Furthermore, we use GroupSHAP to investigate the contribution of single time points within a time series on the model prediction and how excluding time points with the lowest mean average contribution affect the model accuracy.

In our experimental investigations, we demonstrate that models based on image time series data exhibit superior accuracy than the baseline model, which only considers a single time point as input. Furthermore, we show that the explainable machine learning method GroupSHAP effectively facilitates the selection of time points from time series that contribute highly to the result and, thus, leads to improved models.

Our findings can be utilized in new data acquisition methods to control the data acquisition frequency. For instance, data acquisition could be increased during the interval of leaf and shoot development and less during the stage when the head has reached 30% of the expected size, as the development of the plants mainly takes place in the interior of the plant at this time. However, it is important to continuously observe the development from year to year and make adjustments as necessary, considering any variations in the development. To enhance generalization, it is imperative to collect additional data reflecting diverse weather and lighting conditions, as well as additional data stemming from diverse developmental processes concerning the temporal occurrence of growth phases throughout the year, which can subsequently be assimilated into the model framework. Additionally, the findings in the application of cauliflower cultivation can be used to estimate the costs and benefits and determine whether the gain in accuracy justifies acquiring data weeks in advance. Our approach is adaptable and can be extended to other plant varieties or analogous time series analysis tasks.

## Data Availability

Publicly available datasets were analyzed in this study. This data can be found at: https://phenoroam.phenorob.de/geonetwork/srv/eng/catalog.search#/metadata/cb328232-31f5-4b84-a929-8e1ee551d66a.
